# Rapid measures of user’s adherence to vaginal drug products using attenuated total reflectance Fourier transform infrared spectroscopy (ATR-FTIR) and multivariate discriminant techniques

**DOI:** 10.1371/journal.pone.0197906

**Published:** 2018-05-25

**Authors:** Oluwatosin E. Adedipe, Terry A. Jacot, Andrea R. Thurman, Gustavo F. Doncel, Meredith R. Clark

**Affiliations:** 1 CONRAD, Eastern Virginia Medical School, Department of Obstetrics & Gynecology, Norfolk, Virginia, United States of America; 2 CONRAD, Eastern Virginia Medical School, Department of Obstetrics & Gynecology, Arlington, Virginia, United States of America; Harvard Medical School, UNITED STATES

## Abstract

**Background:**

The topical HIV prevention (microbicides) field is in acute need of a method to rapidly and objectively measure adherence to product use in clinical trials. Infrared (IR) spectroscopy has been used in many pharmaceutical and forensic applications but has yet to be applied to adherence monitoring. In this study, we report on efforts to test the feasibility of using IR spectroscopy as a means to measure residual active or placebo vaginal product, semen exposure and vaginal insertion from a single swab.

**Methods:**

A portable IR spectrometer equipped with diamond attenuated total reflectance (ATR) was used to capture spectra of unused vs. vaginally-used swabs, vaginal swabs containing semen, and vaginal swabs to which either tenofovir-containing or matching placebo products (vaginal gel or insert) were added. Spectral data obtained from swabs placed directly on the spectrometer were divided into calibration and testing sets for developing and validating discriminant models set up to provide yes/no predictions of: vaginal vs. non-vaginal use, presence vs. no presence of each test product, and presence vs. no presence of semen. Further validation of models was performed using vaginal swabs collected from a clinical study evaluating vaginally administered placebo insert formulations.

**Results:**

For each discriminant model developed to predict vaginal vs. non-vaginal use, presence vs. no presence of each test product, and presence vs. no presence of semen, classified validation samples not included in the model development were correctly identified into their respective classes with minimal prediction error. Clinically obtained vaginal swabs collected 15–60 minutes after placebo insert use were also correctly identified, further validating the models.

**Conclusion:**

Our findings demonstrate the proof of concept that IR spectroscopy can be a method for rapid detection and characterization of microbicide products and biological fluids present in vaginal swabs. This novel method has potential to support real-time, on-site adherence monitoring in clinical or field settings.

## Introduction

Development of topical microbicides for the prevention of HIV acquisition or other sexually transmitted infections (STIs) has been one of the focal biomedical intervention efforts dedicated to stemming the HIV pandemic [1]. However, several trials have reported poor adherence and, consequently, low effectiveness [2–4]. Additionally, a significant challenge for the microbicides field is the lack of a real-time, objective method of evaluating adherence to product use and study protocol compliance [5–10]. Many studies have reported on difficulties and challenges in measuring adherence in microbicide trials [2–4, 10–13]. Currently, methods for measuring adherence are broadly divided into subjective and objective measures. Subjective measures, including participant self-reports and visual inspections of returned product, are reliant on the observations or reports of clinicians, trial participants, and others involved in the study and thus are prone to error due to misreporting (either intentionally or unintentionally) and/or misinterpretation [2,10]. An objective adherence measure, on the other hand, is independent of operator bias and does not involve self-report from the participant. These may include pharmacologic measures, such as drug levels detected in biological samples or returned used products (e.g., residual drug levels in vaginal rings) [14–16], or biomarkers whose presence or absence indicate that a biological or pharmacological process has occurred in response to a drug [16]. Both of these types of objective adherence measures, however, are inherently constrained by the limitation that they may only be applied to the active drug arm of a clinical study. Moreover, they typically require highly technical, time-intensive and cost-restrictive methodologies such as LC/MS/MS. Hence, there is a need for new methods, preferably conducted in real-time, to accurately measure adherence to product use—be it active or placebo—in clinical trials.

One potential method that would allow rapid detection and characterization of pharmaceutical formulations (active and placebo) and other biological fluids (e.g., semen) is proposed herein with the use of attenuated total reflectance Fourier transformed infrared (ATR FT-IR) spectroscopy for evaluation of vaginal swabs. IR spectroscopy has been applied to address various biomedical applications [17–25], as it is capable of providing, in a matter of seconds, detailed chemical, biochemical, and physical information regarding cells, tissues and biological systems. Recent advances in IR spectroscopy have also allowed collection of spectral data of both solid and liquid samples with little or no sample preparation with the use of ATR spectra collection technique, enabling in situ measurement of biological specimens. Therefore, ATR FT-IR spectroscopy presents a promising tool for many preclinical and clinical applications. However, to date, it has not been exploited as a tool for measuring adherence to pharmaceutical product use in clinical settings.

In this paper, we report the results of a collection of in vitro and in vivo studies designed to investigate the applicability of ATR FT-IR coupled with discriminant models as an adherence measurement tool to confirm the use of vaginal products in microbicide clinical trials. Experiments were set up specifically to determine whether FT-IR could be used to detect and differentiate 1) vaginally inserted swabs from non-vaginally used swabs, 2) the presence or absence of semen on vaginal swabs, 3) the presence or absence of active or placebo microbicide products on vaginal swabs, and 4) feasibility of using FT-IR methodology in a clinical study.

## Materials and methods

Dry double head (Rayon) swabs (StarplexTM Scientific Inc.) were purchased from Fisher Scientific. Universal hydroxyethylcellulose (HEC) placebo gel and tenofovir (TFV) 1% gel were obtained from CONRAD clinical supply inventories, prepared as previously reported [26]. TFV (40 mg) and matching placebo vaginal inserts, all proprietary pharmaceutical products developed by CONRAD were obtained from CONRAD clinical and preclinical supply inventories. Vaginal Fluid Simulant (VFS) was prepared according to the procedure described by Owen and Katz [27]. Samples used for developing the chemometric models were vaginal swabs provided by volunteers, vaginal swabs spiked with semen donated by volunteers, and vaginal swabs obtained at various time points post vaginal insert use (CONRAD D15-134).

### Ethics statement

Vaginally-inserted swabs were obtained from healthy women under a protocol/consent approved by the EVMS Institutional Review Board (IRB), (09-09-FB-0175). Vaginal swabs, collected at various times post-vaginal administrations of a placebo insert, were obtained from the CONRAD D15-134 study (ClinTrial #NCT02534779). The study assessed the disintegration/disappearance time, safety, and acceptability of placebo vaginal inserts used by generally healthy women between the ages of 18–50. The protocol and consent form was approved by the Chesapeake IRB (Pro00012885). Semen samples were provided by healthy, normozoospermic donors under an EVMS IRB approved protocol (13-02-FB-0031). All volunteers/participants provided a written, informed consent.

### FTIR equipment, spectra acquisition and chemometrics analysis software

FTIR spectra data (wavenumber range 4000–650 cm^-1^) for each sample swab was obtained by directly placing swab on a portable Agilent Cary 630 FTIR Spectrometer equipped with a Diamond Head accessory and Microlab PC software run from a dedicated computer laptop. Multivariate data analysis of spectra was performed using The Unscrambler X software Version 10.3 (Camo Smart, Woodbridge, NJ, USA) and Grams IQ software (Thermo Fisher Scientific Inc.). Discriminant models were developed using Soft Independent Modeling of Class Analogies (SIMCA) and Mahalanobis Distance by Principal Component Analysis with Residual (MD/PCA/R).

### Experimental design

In vitro and in vivo experiments were carried out to investigate the applicability of FTIR method for rapid detection and characterization of microbicides and biological fluids present in vaginal swabs. Separate discriminant models were developed and tested to predict presence of vaginal fluids versus other fluids/simulants, presence or absence of semen, presence or absence of placebo HEC gel or insert, and presence or absence of TFV gel and active insert. Discriminant models such as SIMCA can work with as few as 10 samples per class with no restriction on the number of measurement variables [28]. The sample size used in each of the models developed varies for each class with at least 20 samples per class model. Vaginal swab samples collected from donors who did not use drug or other vaginal products were selected for model development. Details about the experimental design and discriminant model development for each of the experiments are described as follows:

### Vaginally-used vs. control swabs

Spectra of vaginally-used swabs and control swabs (i.e., not vaginally inserted) were used in model development and validation. The control swabs were prepared by adding 100 μL of deionized water or VFS to dry rayon swabs, and then air dried for 30 minutes before FTIR spectra acquisition. A total of 150 spectral data consisting of 60 vaginally inserted swabs, 30 spectra of unused dry rayon swabs (Control- Dry), 30 spectra of swabs dipped in deionized water (Control-water), and 30 spectra of swabs dipped in VFS (Control-VFS) were acquired by FTIR. 75 spectra were randomly selected from the spectral data and used as the calibration training set to develop SIMCA and MD/PCA/R discriminant models. The raw spectra and spectra preprocessed using Savitzky-Golay first derivative method [29] were used to develop models using the full IR spectra region of 4000–650 cm^-1^, the broad IR region of 1740–1140 cm^-1^, and a restricted IR region of 1685–1485 cm^-1^. A separate testing set (n = 75) was then used to test if FTIR coupled with the discriminant model can be used to differentiate and correctly classify a specimen with vaginal secretions from the specimen with no vaginal secretions.

### Presence vs. absence of semen

A total of 100 spectra data consisting of 32 spectra of vaginal swabs with no semen (baseline), 40 spectra of simulated vaginal swabs containing semen and 28 spectra of rayon swabs dipped in semen were used in model development and validation. Vaginal swabs containing semen were prepared by dipping vaginal swabs into semen samples. Vaginal swabs and rayon swabs coated with semen were allowed to air dry for 30 minutes before FTIR spectra acquisition. Three quarter spectra from each group were randomly selected for use in the calibration set (n = 76) and discriminant model development based on SIMCA methodology. Models were developed with the raw spectra using full IR region (4000–650 cm^-1^), and spectral data restricted to IR regions of 1800–650 cm^-1^ and 1700–1130 cm^-1^. The remaining 24 spectral data were used to validate and test if the models can identify and correctly classify vaginal swabs containing semen (vaginal secretion + semen), semen only (rayon + semen) from the “baseline” vaginal swabs.

### Presence vs. absence of placebo product (HEC gel and placebo insert)

Solutions of universal HEC gel and placebo insert were prepared by dissolving or diluting the formulations at different concentrations (0.5, 0.25, 0.05, and 0.01 g/mL) in deionized water. A 100 μl aliquot of each prepared solution was then added by pipette to vaginal swabs. 100 spectra of vaginal swabs spiked wih HEC placebo gel of varying concentrations, 48 spectra of vaginal swabs spiked with varying concentrations of placebo insert, and 30 spectra of vaginal swabs with no placebo formulation added (i.e., baseline) were captured. Two-thirds of the spectral data (n = 66 for HEC placebo gel and n = 36 for placebo insert) were randomly selected and used in developing discriminant models for each dosage form. Chemometric models for discriminating between vaginal swabs containing HEC placebo gel from baseline were developed using SIMCA method with the full IR region (4000–650 cm^-1^) and restricted IR spectra regions of 1480–780 cm^-1^ with both the raw spectra and spectra preprocessed using Savitzky-Golay first derivative transformation method. A validation set (n = 60) consisting of the remaining 30 spectral data of HEC placebo gel and 30 spectra of vaginal swabs (i.e., no placebo gel) was used to validate and test if models can correctly classify vaginal swab containing placebo gel from baseline.

MD/PCA-R discriminant models for separation of vaginal swabs containing placebo insert from the baseline swabs were developed with the raw IR spectra using the full IR region (4000–650 cm^-1^), IR region of 1700–780 cm^-1^ and IR region restricted to 1200–780 cm^-1^. The placebo insert model developed was first validated with in vitro samples (n = 24) consisting of the remaining 12 spectral data of placebo insert-spiked swabs and 12 spectra of baseline vaginal swabs (i.e., no placebo insert). The placebo insert model was further validated with vaginal swabs collected from 9 participants in CONRAD-134 clinical study (NCT02534779) in which the FTIR assessor was blinded. The study involved women using a placebo vaginal insert, and vaginal swabs were collected from the participants over a course of three- time points: 1) before vaginal administration of the inserts (baseline, denoted as Visit 2 pre or V2-Pre), 2) after 15–60 minutes of administering the placebo insert (V2-Post), and 3) after 24–48 hours of placebo insert administration (V3-Post).

### Presence vs. absence of TFV (TFV gel and TFV insert)

Spectra of TFV, the active pharmaceutical ingredient (API) in the gel and insert formulation, were acquired to determine its spectral fingerprint. Solution of TFV-containing gel and insert formulations of varying concentrations (4, 2,1,0.5 and 0.25 g/mL) were prepared by dissolving in deionized water. A 100 μl aliquot of each prepared solution was then added by pipette to vaginal swabs. Spectra of vaginal swabs containing varying concentrations of active product (45 spectra for TFV gel, 45 spectra for TFV insert), placebo product (45 spectra for HEC gel, 45 spectra for placebo insert), or no product (10 spectra for baseline) were captured. Two thirds of the spectral data for each of TFV dosage form were randomly selected and used in developing MD/PCA-R discriminant models for TFV gel, TFV inserts, placebo gel and placebo insert. Models for separation of vaginal swabs containing either active gel or insert from vaginal swabs containing placebo product were developed with the raw spectra and spectra pre-processed using Savitzky-Golay first derivative method using the full IR region (4000–650 cm^-1^), and restricted IR spectra regions of 1120–890 cm^-1^. A validation set (n = 30) was created, consisting of 10 spectral data for each group of vaginal swabs containing active product (TFV gel or inserts), spectra of vaginal swabs containing placebo gel or inserts, and spectra of baseline swabs.

## Data analysis and model development

Different calibration models were developed using raw spectra and spectra pre-processed using either Savitzky-Golay first or second derivative methods to determine if spectral pretreatment will improve the interpretation of the spectra, integrity, and the applicability of the calibration models [29]. Because IR spectra may contain subtle information which is not visible as individual peaks, the full IR region (4000–650 cm^-1^) and broad IR wavelength regions were selected to construct models capable of discrimination between test groups. Before model development, principal component analysis (PCA) was carried out to observe any clustering or separation in the data set, and subsequently, SIMCA or MD/PCA-R approach was used to build prediction models. Detailed descriptions of both PCA and PLS discriminant modeling approaches have been reviewed extensively in chemometrics literatures [29–34].

## Results

### Discrimination of vaginally inserted swabs from control swabs

**Fig 1:**
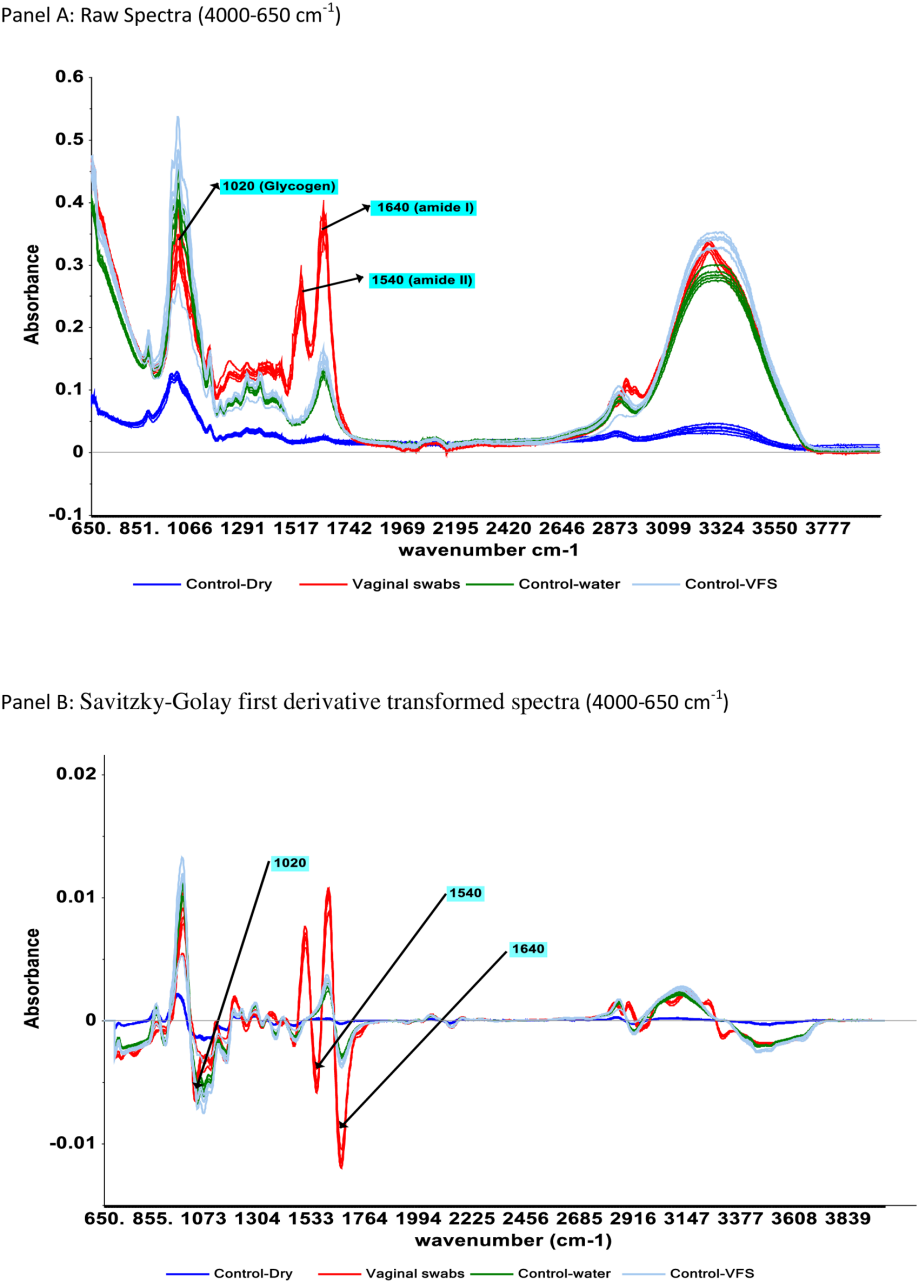
Representative IR raw (panel A) and Savitzky-Golay first derivative transformed spectra (panel B) of vaginally inserted swabs, control-dry (rayon swabs), control–water (rayon dipped in water) and control-VSF (rayon dipped in vaginal fluid simulant).

Raw spectra of vaginally inserted and control swabs used for model development are presented in Fig 1 (panel A). Most of the spectra looked alike, except for the spectra of the dry rayon swabs that had lower absorption intensities all across the IR spectra region. Spectra of vaginal swabs, and spectra of swabs dipped in deionized water or VFS appeared to be similar in appearance and showed broad peaks centered around 1640 cm^-1^ and 1540 cm^-1^ and 1020 cm^-1^.

**Fig 2:**
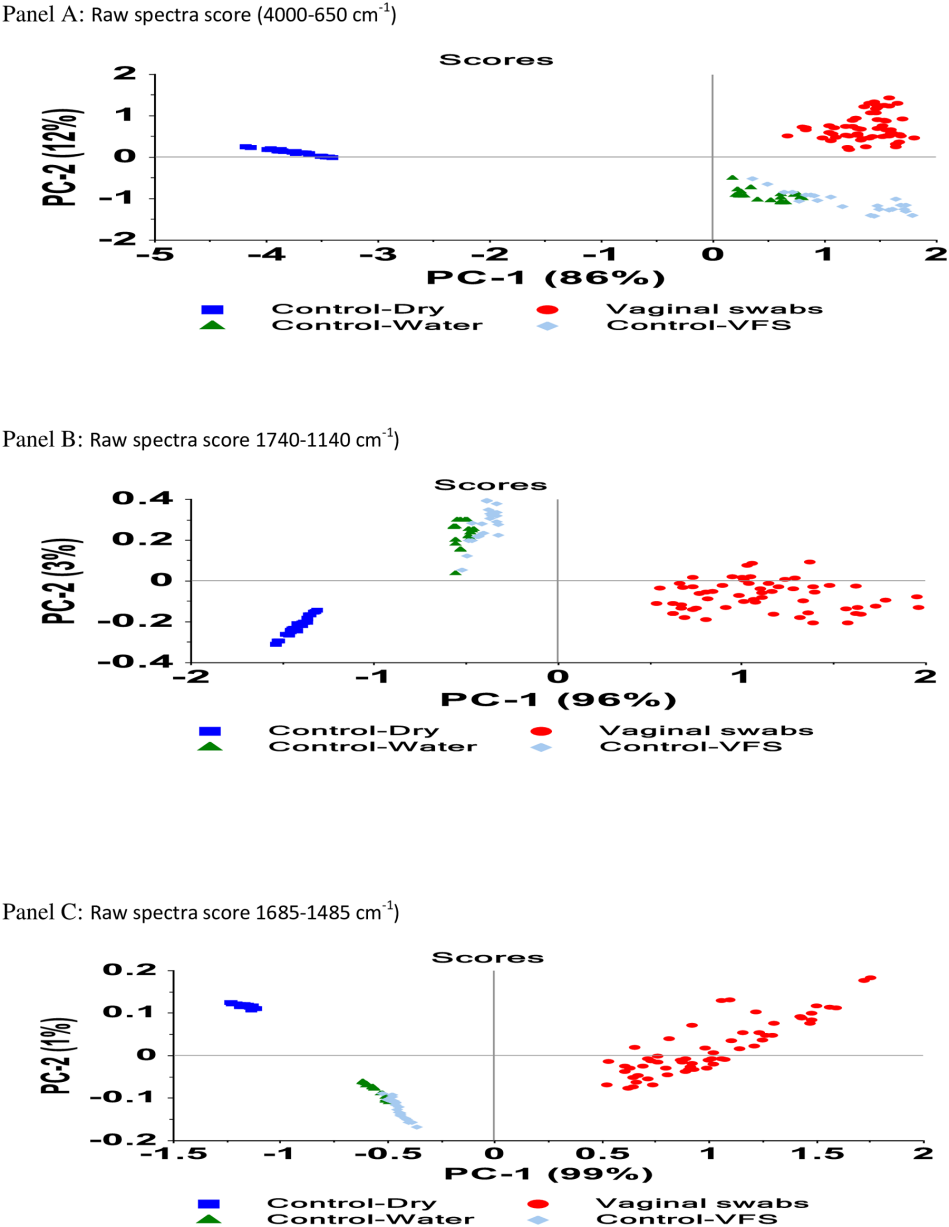
PCA score plots showing separation of vaginally inserted swabs and various control swab conditions (dry, or treated with water or VFS) using raw spectra (panel A), IR region of 1740–1140 cm^-1^(panel B), and IR region of 1685–1485 cm^-1^(panel C).

Principal component analysis (PCA) of spectra was carried out to obtain an overview of the data and check for underlying patterns or relationships and outliers in the specimen set. PCA revealed clustering of similar samples and clear separation of sample groups that were not similar (Fig 2). The first two principal components (2 pc’s) explained 98, 99 and 100% spectral data variation respectively for models developed using the full IR spectra region of 4000–650 cm^-1^, IR region of 1740–1140 cm^-1^, and IR region of 1685–1485 cm^-1^.

SIMCA models developed with raw spectra or spectral data treated with first derivative method, and tested at 5% significance level performed well in the identification of vaginal swabs not included in calibration set, with accuracy ranging from 97–100% with low error (Table 1) The count of prediction false negatives and false positives is expressed as “Type I” and “Type II” error respectively. Type I error associated with models ranged from 0–3%, while “Type II error was 0 for all models. Spectral data pretreatment with first derivative transformation (Fig 1, panel B) did not significantly improve model predictions as shown in Table 1.

**Table 1:** Summary of classification models developed for identification of vaginal swabs.

Model	Class	npc's^a^	Testing set (n = 75)^b^	Samples correctly classified (%)	Type I error (%)^c^	Type II error (%)^d^
Raw spectra 4000–650 cm^-1^	**Vaginal swabs**	**3**	**30**	**100**	**0**	**0**
	Control-Dry	2	15	100	0	0
	Control-Water	3	15	100	0	0.1
	Control-VFS	2	15	100	0	0.3
Raw spectra 1470–1170 cm^-1^	**Vaginal swabs**	**3**	**30**	**100**	**0**	**0**
	Control-Dry	2	15	100	0	0
	Control-Water	2	15	100	0	0
	Control-VFS	2	15	83	17	0.1
Raw spectra 1685–1485 cm-^1^	**Vaginal swabs**	**2**	**30**	**97**	**3**	**0**
	Control-Dry	3	15	94	6	0
	Control-Water	2	15	100	0	0
	Control-VFS	2	15	92	8	0.1
Savitzky-Golay 1st der. spectra 4000–650 cm-^1^	**Vaginal swabs**	**5**	**30**	**100**	**0**	**0**
	Control-Dry	3	15	100	0	0
	Control-Water	3	15	100	0	0.1
	Control-VFS	3	15	92	8	0.2
Savitzky-Golay 1st der. spectra 1470–1170 cm^-1^	**Vaginal swabs**	**3**	**30**	**97**	**3**	**0**
	Control-Dry	3	15	100	0	0
	Control-Water	3	15	100	0	0.1
	Control-VFS	2	15	92	8	0.3
Savitzky-Golay 1st der. spectra 1685–1485 cm^-1^	**Vaginal swabs**	**3**	**30**	**100**	**0**	**0**
	Control-Dry	3	15	100	0	0
	Control-Water	3	15	100	0	0.1
	Control-VFS	3	15	92	8	0.2

^a^Optimum number of principal components (npc’s) used in models development

^b^Testing set (75) consist of 30 vaginal swabs and 45 control swabs

^c^Type I error (%) was calculated based on number of predicted false negatives for each model

^d^Type II error (%) was calculated based on number of predicted false positives for each model

The result of the PCA and SIMCA models indicates that classification models capable of discriminating different sample groups could be developed using any one of these IR spectral regions. However, because of its fewer principal component used for model, the full IR raw spectra model was selected as the best to discriminate between vaginally inserted swabs and various control swabs and was later set up on the FTIR equipment to provide a yes/no prediction for new samples, thereby providing additional validation as showed in S1 Table. Models were developed with the Grams software using the full IR spectra region of 4000–650 cm^-1^. The model was 100% correct in classifying the validation specimens to their respective group.

### Discrimination of presence vs. absence of semen on vaginal swabs

**Fig 3:**
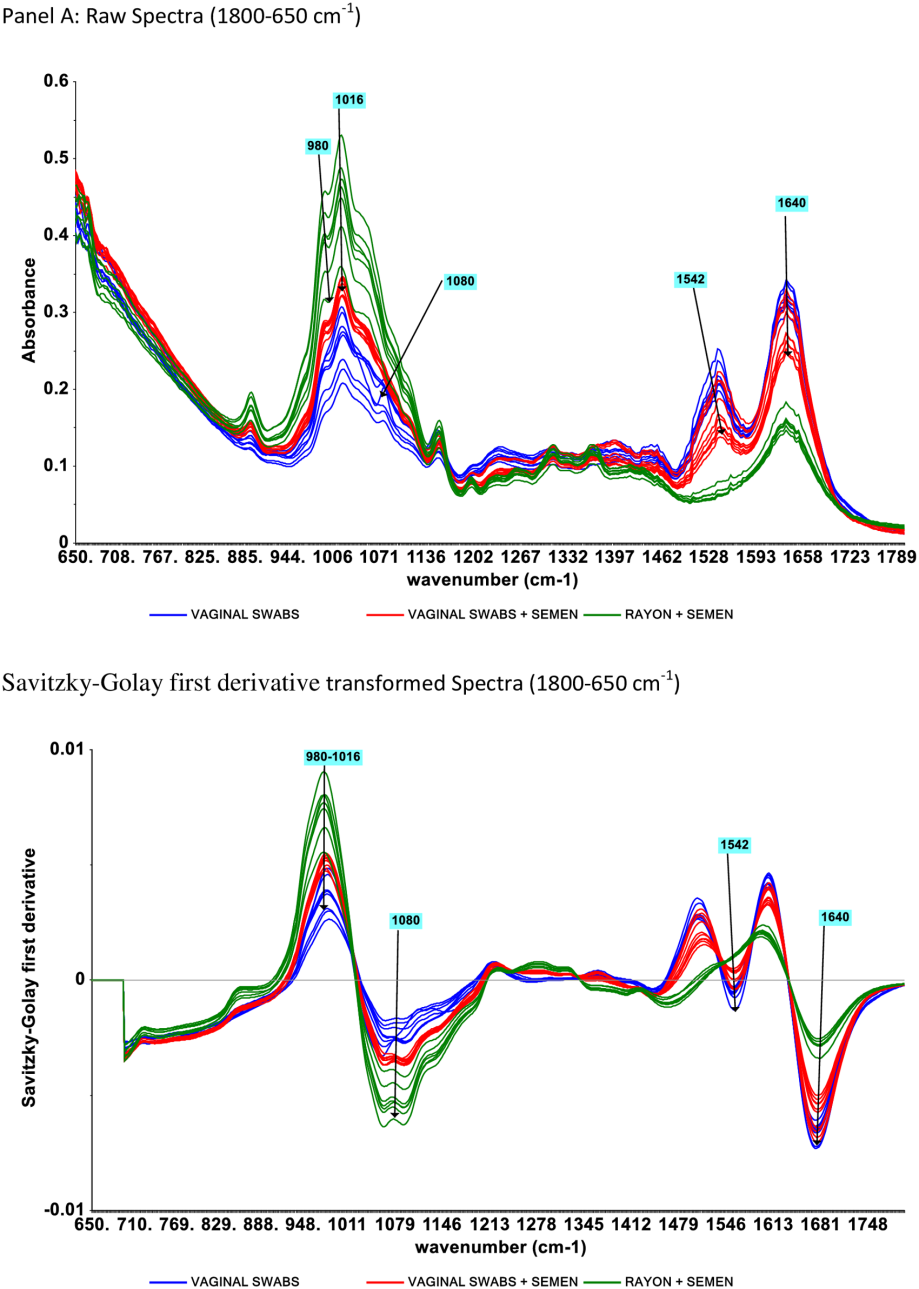
Representative raw infrared spectra (panel A) and Savitzky-Golay first derivative transformed spectra (panel B) of vaginal swabs, vaginal swabs + semen and rayon + semen.

Apart from changes in absorption intensities in the IR fingerprint region (900–1800 cm^-1^), spectra of vaginal swabs with and without semen appeared similar (Fig 3). Spectra of samples containing semen showed higher absorption intensities than the baseline at IR peak region centered at 1016 cm^-1^. Peak centered on IR region of 1080 and 1542 cm^-1^ also showed clear difference between samples containing vaginal secretion (baseline and vaginal swabs containing semen) from semen only swabs (rayon dipped in semen) as revealed in Fig 3.

PCA revealed clusetering and clear separation of groups for models developed using the full IR region or models restricted to IR region of 1800–650 cm-^1^ and 1700–1130 cm^-1^. However, PCA score plots indicates using IR region of 1800–650 cm^-1^ may produce the best model that could achieve clear separation of samples of vaginal swabs + semen from vaginal swabs with no semen (baseline) and rayon swab + semen only (S1 Fig panel B). Table 2 summarizes the results of the prediction of SIMCA models tested at 5% significance level. SIMCA models developed with raw spectra and spectral data pre-treated with first derivative transformation method performed well in the identification of new samples. Although, models developed using the full IR region or the restricted IR region of 1700–1130 cm^-1^ were 100% accurate in assigning the validation samples to their respective groups, results showed that such model may be prone to a high risk of identifying false positives. The results showed that models developed using the 1800–650 cm^-1^ IR region is the best. The models were 100% correct in assigning the validation samples to their respective groups with no false negatives or false positives (Table 2).

**Table 2:** Summary of classification models tested at 5% significance level for identification of vaginal swabs (baseline) from vaginal swabs containing semen and rayon swabs containing semen only.

Model	Class	npc's^a^	Testing set (n = 24)^b^	Samples correctly classified (%)	Type I Error (%)^c^	Type II Error (%)^d^
Raw 4000–650 cm^-1^	Baseline	4	8	100	0	0
	Vaginal swabs + Semen	4	10	100	0	4
	Rayon + Semen	3	6	100	0	0
Raw 1800–650 cm^-1^	Baseline	4	8	100	0	0
	Vaginal swabs + Semen	4	10	100	0	0
	Rayon + Semen	2	6	100	0	0
Raw 1130–1700 cm^-1^	Baseline	4	8	100	0	0
	Vaginal swabs + Semen	2	10	100	0	8
	Rayon + Semen	3	6	100	0	4
Savitzky-Golay 1st der. 4000–650 cm^-1^	Baseline	4	8	100	0	0
	Vaginal swabs + Semen	4	10	100	0	0
	Rayon + Semen	2	6	100	0	0
Savitzky-Golay 1st der. 1800–650 cm^-1^	Baseline	4	8	100	0	0
	Vaginal swabs + Semen	4	10	100	0	0
	Rayon + Semen	2	6	100	0	0
Savitzky-Golay 1st der. 1800–1130 cm^-1^	Baseline	4	8	100	0	4
	Vaginal swabs + Semen	2	10	100	0	0
	Rayon + Semen	3	6	100	0	0

^a^Optimum number of principal components (npc’s) used in the model development

^b^Testing set (75) consist of 8 spectra of vaginal swabs (Baseline, no semen), 10 spectra of vaginal swab + semen, and 6 spectra of rayon swabs containing semen

^c^Type I error (%) was calculated based on number of predicted false negatives sample per model

^d^Type II error (%) was calculated based on number of predicted false positives samples per model

### Discrimination of presence vs. absence of placebo product (vaginal gel or insert)

Raw spectra (1700–1480 cm^-1^) of vaginal swabs (baseline) and vaginal swabs containing different concentrations of HEC placebo gel is presented in S2 Fig. The spectra look very much alike with broad peaks, making assignment difficult. PCA revealed a clear separation between vaginal swabs with no HEC gel (baseline) swabs and vaginal swabs containing HEC placebo gel (S3 Fig). SIMCA classification models developed with raw spectra and spectral data pre-treated with first derivative transformation method performed well in the identification of new samples with accuracy ranging from 93–97% with low error. Table 3 summarizes the results of the predictions of different SIMCA models developed using the full IR spectra region of 4000–650 cm^-1^ and the selected IR region of 1700–1480 cm^-1^. Using the raw spectra and the spectra pre-treated with first derivative transformation at 5% significance level of testing, Type I errors associated with all calibration models developed with identifying new vaginal swabs containing HEC placebo gel ranged from 3–7%, while Type II error ranged from 0–3%.

**Table 3:** Summary of classification models developed for identification of vaginal swabs containing HEC placebo gel tested at 5% significance level.

Model	Class	npc's^a^	Testing set (n)^b^	Vaginal swabs with HEC gel classified correctly (%)	Type I error (%)^c^	Type II error (%)^d^
Raw (4000–650 cm-^1^)	Vaginal swab + HEC	2	60	97	3	3
Raw (1700–1480 cm-^1^)	Vaginal swab + HEC	2	60	97	3	0
Savitzky-Golay (4000–650 cm-^1^)	Vaginal swab + HEC	2	60	93	7	3
Savitzky-Golay (1700–1480 cm-^1^)	Vaginal swab + HEC	2	60	97	3	0

^a^Optimum number of principal components (npc’s) used in model development

^b^Testing set (n = 60) consist of 30 spectra of vaginal swab containing HEC placebo gel + 30 vaginal swabs (baseline, no gel)

^c^Type I error (%) was calculated based on number of predicted false negatives for each model

^d^Type II error (%) was calculated based on number of predicted false positives for each model

**Fig 4:**
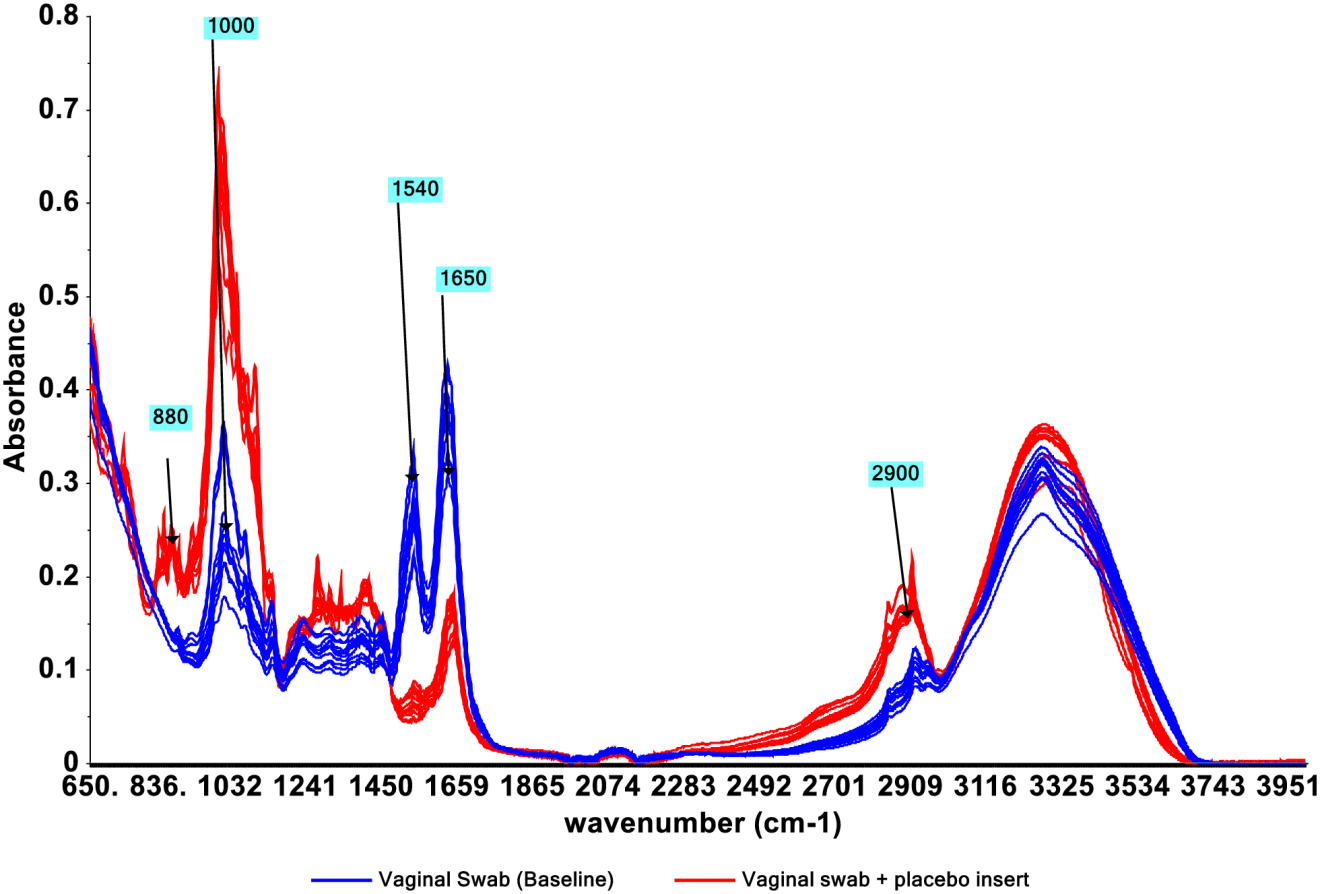
Raw spectra(4000–650 cm^-1^) of vaginal swabs (baseline, no placebo insert) and vaginal swabs containing placebo insert.

In the case of placebo inserts, five distinct peaks centered at 880, 1000, 1540, 1650 and 2900 cm^1^ clearly differentiated vaginal swabs (baseline) from vaginal swabs containing placebo insert as shown in Fig 4.

PCA of spectral data revealed a clear separation of vaginal swabs containing placebo insert from the baseline (S4 Fig, panel A) and also clustering of vaginal swabs containing similar concentration of placebo insert (S4 Fig, panel B). The first two principal components (2 pc’s) explained 93%, 97% and 99% spectral data variation, respectively, for models developed with the raw IR spectra using the full IR region (4000–650 cm^-1^), IR region of 1700–780 cm^-1^ and IR region restricted to 1200–780 cm^-1^. The result of the PCA indicates that classification models capable of discriminating between vaginal swabs containing placebo and vaginal swab (baseline) could be developed using any one of these IR regions. MD/PCA-R discriminant models was developed using Grams software, the model developed with raw spectra and using the restricted IR region of 1200–780 cm^-1^ region performed well in the identification of new in vitro samples not included in model development. All validation samples were correctly classified into their respective groups with 100% accuracy (S2 Table). The feasibility of using FTIR methods coupled with discriminant model was further validated using vaginal swabs collected from a clinical study of vaginally adminstered placebo inserts (CONRAD D15-134). The FTIR model was 100% accurate in identifying correctly all baseline swabs (V2-Pre) and vaginal swabs containing placebo insert collected 15–60 minutes (V2-Post) after insert administration. 80% of swabs, collected 24–72 hours (V3-Post) after application of vaginal inserts were correctly identified as presented in Table 4.

**Table 4:** FTIR predictions summary for placebo insert study (CONRAD D15-134).

Time (hrs)	Visit	Number of participants	% samples correctly identified
0	V2-Pre (baseline)^a^	9	100
0.25–1	V2-Post^b^	9	100
24–72	V3 post^c^	8	80

^a^ baseline swabs collected at clinical site prior to insert administration

^b^ swabs collected at clinical site 15–60 minutes after insert administration

^c^ Self swabs collected at home 24–72 hours after insert administration

### Vaginal swabs containing Tenovofir (TFV) gel/inserts

**Fig 5:**
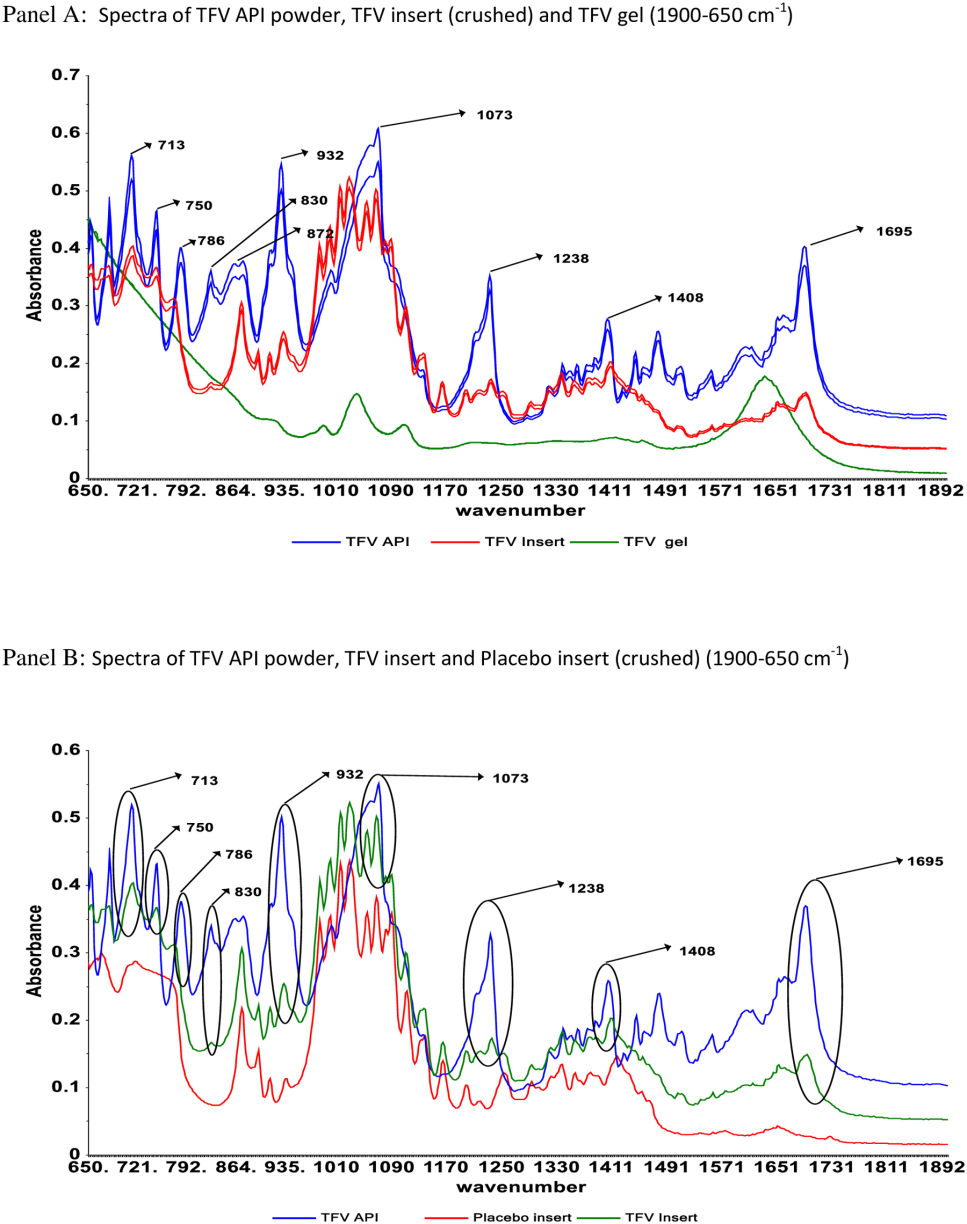
TFV API spectra profile and its corresponding functional peaks in spectra of TFV gel and insert formulations.

Fig 5 shows the spectra of TFV powder, the active pharmaceutical ingredient (API) in the TFV insert and gel formulations (also shown in Fig 5 for comparison). The dominant peaks of TFV API are found in the IR fingerprint region centered at 1696, 1408, 1073, 932, 830, 786, 7160 and 713 cm^-1^.

Spectra of baseline vaginal swabs and vaginal swabs containing varying concentrations of TFV gel and spectra of vaginal swabs containing placebo gel are presented in S5 Fig (panel A). There are two distinct broad bands in the IR fingerprint region (850–1200 cm^-1^) with peaks centered at 932, and 1040 cm^-1^ that showed visual differences among spectra of TFV gel at different concentrations in vaginal swabs. Absorption intensity of these peaks correlates with the concentration of TFV gel in vaginal swabs as shown in S5 Fig (panel B). Peaks centered at 830, 932 and 1073 cm^-1^ are dominant features separating vaginal swabs containing active gel from placebo gel or baseline swabs, as revealed in S5 Fig, Panel B. PCA score plots of model developed with the raw spectra using the full IR region, and IR region restricted to 1800–650 cm^-1^ and 930–830 cm^-1^ for separation of vaginal swabs containing active gel from placebo or baseline are presented in S6 Fig. PCA score plots revealed that IR region of 930–830 cm^−1^ will give the best model for clear and unambigious separation of vaginal swabs containing active TFV gel from HEC placebo or baseline. This is an indication that FTIR can serve as a tool to separate between vaginal swabs containing TFV gel and baseline or placebo swabs (no active) or estimate the concentration of active gel in vaginal swabs.

A similar PCA result was observed for models designed using IR region of 1120–890 cm^-1^ for separation of vaginal swabs containing TFV inserts and matching placebo inserts respectively (data not shown). The validation results of MD/PCA-R discriminant models designed respectively for identification and discrimination of vaginal swabs containing TFV active inserts from vaginal swabs containing placebo inserts or baseline vaginal swabs is presented in Table 5. All validation samples were correctly classified into their respective groups with 100% accuracy.

**Table 5:** Validation of discriminant models developed for identification of vaginal swabs (baseline) and vaginal swabs containing placebo or TFV active inserts.

	IS PLACEBO INSERT PRESENT?		IS TFV ACTIVE INSERT PRESENT?	
Validation sample	Match^a1^	Mahalanobis Distance (M.D.) ^b^	Match^a2^	M.D. ^b^
PLACEBO SWAB	YES	1.59	No	55.35
PLACEBO SWAB	Yes	0.74	No	34.04
PLACEBO SWAB	YES	1.07	No	87.84
PLACEBO SWAB	Yes	0.97	No	25.60
PLACEBO SWAB	YES	1.05	No	11.55
PLACEBO SWAB	YES	1.08	No	14.69
PLACEBO SWAB	Yes	0.84	No	13.58
PLACEBO SWAB	Yes	0.90	No	15.26
PLACEBO SWAB	Yes	0.81	No	15.13
PLACEBO SWAB	YES	2.79	No	33.72
TFV SWAB	No	1574.39	YES	2.81
TFV SWAB	No	1375.73	YES	1.39
TFV SWAB	No	1787.60	Yes	0.92
TFV SWAB	No	3224.16	YES	1.02
TFV SWAB	No	2624.03	Yes	0.93
TFV SWAB	No	2763.74	Yes	0.78
TFV SWAB	No	2047.04	Yes	0.97
TFV SWAB	No	2156.48	YES	2.23
TFV SWAB	No	1373.56	Yes	0.94
TFV SWAB	No	385.53	Yes	0.81
BASELINE SWAB	No	11.12	No	13.90
BASELINE SWAB	No	7.02	No	12.66
BASELINE SWAB	No	10.84	No	15.96
BASELINE SWAB	No	17.47	No	22.11
BASELINE SWAB	No	9.58	No	17.45
BASELINE SWAB	No	15.59	No	14.84
BASELINE SWAB	No	13.55	No	13.36
BASELINE SWAB	No	12.31	No	13.62
BASELINE SWAB	No	16.75	No	18.66
BASELINE SWAB	No	14.09	No	14.24

^a^Yes/no prediction based on model question: Is placebo (^a1^) or TFV (^a2^) present in vaginal swab?

^b^A specimen with M.D. greater than 3 is classified as NO for presence of TFV or placebo insert, while a specimen with M.D of less than 3 is classified as a Yes member

## Discussion

### IR spectroscopy coupled with multivariate data analysis

Traditionally, IR spectral data are analyzed by assignment of distinct peaks to a single property (specific chemical markers) using a univariate approach. This type of analysis is difficult for complex biological systems that are best described using multiple factors that often interfere and are closely related to each other. Spectroscopists have realized the need to use multivariate data analysis (MVDA) techniques when dealing with spectral data of complex materials [21]. MVDA methods are capable of extracting information from spectra by allowing the use of spectra data points over a broad range of wavelengths. MVDA methods have been proven to be superior and are much better able to obtain useful qualitative and quantitative information from spectra than classical or univariate statistical methods[28–34]. Our main goal was to determine unambiguously if a vaginal swab sample contained vaginal secretions, semen or drug products (active or placebo) based on calibration or training set of similar samples. We therefore employed SIMCA and MD/PCA-R, a technique similar to SIMCA methodology, to assess feasibility of using IR spectroscopy coupled with discriminant models as an objective adherence measurement tool to confirm the use of vaginal products in microbicide clinical trials.

The SIMCA approach to classification is one of the main tools in supervised pattern recognition. It is based on using separate bilinear modeling of a true data class, and often the individual data class models are principal component models. In SIMCA-classification, the test result carried out quantifies the risk that a particular object falls outside a specific model even if it truly belongs. In the SIMCA classification setup, two different types of error are recognized: (1) Type 1 error, or “false negative”, which tends to reject a specimen that belongs to the classification group, and (2) Type II error, or “false positive”, which tends to accept a sample as a member when the specimen does not fall into the classification group. Classification results are often evaluated with varying significance levels and it is used to check the distance of a sample to a model. A low significance level of 5% used in our studies means that the model will have 5% risks that a sample falls outside a class group, even if it truly belongs to the group [34].

### Detection of presence or absence of vaginal secretions

For a vaginal swab that may be self-collected by a study participant for use as a potential adherence measure in a clinical trial, it is important to be able to confirm that the swab was in fact vaginally inserted. Therefore, we evaluated the feasibility of developing a discriminatory model to differentiate a vaginally used swab from unused control swabs of various preparations (dry, exposed to water, VFS).

Studies by different groups have identified major vibrational bands for vaginal mucus to multiple IR spectra wavelength bands [35–36]. A typical ATR FT-IR spectrum of vaginal secretions (as shown in S1 Fig, (panel A) exhibited four dominant IR peaks around 3274 cm^-1^ (amide A), 1640 cm^-1^ (amide I), 1542 cm^-1^ (amide II) and 1030–1126 cm^-1^ (glycogen), in addition to some smaller, medium or weak peaks. These peaks were previously identified as methylene stretches of vaginal mucosa (2923 cm ^-1^), methyl bending of proteins (1457 and 1406 cm ^-1^) and nucleic acid phosphates (1232 cm ^-1^) [35–36].

From the IR spectra of vaginal swabs compared to those of control swabs, the vibration bands with peak center around 1540 cm^-1^ appear to be the IR peak of interest in separation of the spectra of the swabs. The peak centered around 1540 cm^-1^ is unique to swabs with vaginal secretions. Spectra of vaginal swabs have higher absorption intensity at this peak while it's almost non-existence for spectra of control swabs containing water and VFS (Fig 1). The amide II peak could be associated with proteins present in the vaginal fluid, which could have originated from bacteria, fungi and other multiple sources of proteins [25,35–36]. In a reported study [35], acid phosphatase (AP) was identified to be a protein of interest responsible for the amide I and II peaks in vaginal secretion.

All models developed using the full IR spectra region of 4000–650 cm^-1^, the broad IR region of 1740–1140 cm^-1^, and restricted IR region of 1685–1485 cm^-1^ performed well in discriminating between swabs with vaginal secretions and control swabs as shown in Fig 2 and Table 1. Models developed using the full spectra represent the best model for discriminating between swabs with vaginal secretion and unused swabs (Table 1). Although, all models developed using the full IR spectra region of 4000–650 cm^-1^ or spectra restricted to IR region of 1740–1140 cm^-1^, and IR region of 1685–1485 cm^-1^ all performed equally well, model developed with the full raw IR spectra region of 4000–650 cm^-1^ was picked to be the best. This model was picked because of its low error and number of principle components, coupled with the advantage of using the full IR spectra. The full IR spectra contains many features that are subtle and unknown but may be important in discriminating vaginal swabs from control swabs used in this study or swabs containing other body fluids, biological and chemical products. Spectral data pre-treatment with Savitzky-Golay second derivative transformation did not appear to improve spectra visual differences. Models developed with Savitzky-Golay second derivative transformed spectra therefore were not significantly better than models developed with the unprocessed spectra (Table 1). To assess feasibility of implementing this vaginal swab model analysis into a rapid and real-time adherence measurement tool, a MD/PCA-R discriminant model was developed with the full IR spectra using the Grams software, which was set up to give a YES/NO prediction. The MD/PCA-R discriminant model, which is similar to the SIMCA methodology and will give the same results as SIMCA was chosen over SIMCA for the implementation stage because of FTIR software compatibility issues. The model was subsequently validated using samples that were not included in the calibration set (S1 Table)

### Detection of semen on vaginal swabs

It is also very important to be able to determine if semen is present or not in a self collected swab by a study participant. This piece of information could be very useful especially when dealing with participants that are prone to high risk of HIV infection. Therefore, we evaluated the feasibility of developing a discriminatory model to differentiate a vaginally inserted swab with no semen (baseline) from vaginal swabs containing semen, and swabs containing senem only (rayon dipped in semen, no vaginal secretion).

Semen is an organic fluid that consists of two parts, the cellular part (spermatozoa and in some instances leukocytes and epithelial cells) and the noncellular part (seminal plasma). Semen contains a heterogeneous list of compounds and ions [35]. IR spectra in the fingerprint region (900–1800 cm^-1^) are primarily proteins, with band structure modified by nucleic acid and other components [37–40].

In earlier studies by different groups, semen was found to produce FT-IR spectroscopic bands at 1016, 966, 1657, 1547, 1450, 1400, 1236, 1087, and 1740 cm^-1^ [25,35–38]. The peak at 1016 cm^-1^ was identified to be a distinct signature peak for semen [25]. ATR FT-IR spectra of dried semen (as shown in S1 Fig, panel A) consist of five dominant peaks at IR regions 1625 cm ^-1^ (amide I), 1540 cm ^-1^ (amide II), 1400 (amino acids), 1060 cm ^-1^ (glycoproteins) and 3273 cm ^-1^ (amide A), in addition to a number of smaller peaks at 2941, 1450, 1240 and 980 cm ^-1^. The findings of our study are consistent with a previous report of ATR FT-IR analysis of body fluids [35].

Spectra features that visually separate vaginal swabs with semen from baseline vaginal swabs and semen only swabs were found in IR region 1800–650 cm^-1^. This IR region contained four peaks centered at 1016, 1080, 1547 and 1657 cm^-1^, with the peak centered on 1016 cm^-1^ being the most dominant. Absorption intensity of spectra of vaginal swabs with semen is higher around this region (S1 Fig, panel A). Vaginal swabs containing semen also have higher intensity at peaks centered at 1080cm^-1^ (Fig 3, Panel B). Because semen and vaginal secretions are known to exhibit similar peak conformations across the spectrum [35], the visual differences in peak intensities of semen and vaginal fluid spectra at IR regions of 1016 or 1080 cm^-1^ reported in this study werenot used as primary discriminants between the two groups. IR peak intensities are known to be easily influnced by external factors such as instrumental (temperature changes), environmental (moisture) and materials handling (sampling position and other unknown substances that may be present in samples). Distinction based on the spectral pattern of peaks produced from these body fluid samples is more reliable, consistent and reproducible. However, using a combination of the peak strengths, patterns and frequencies exhibited within the fingerprint region enables these two body fluids to be distinguished from one another [35].

Although models could be developed using the full IR spectra, models developed using the restricted IR region of 1800–650 cm^-1^ (S1 Fig, panel B) provided better predictions with no false negative and false positives predictions, as shown in Table 2. Spectral data pre-treated with Savitzky-Golay first derivative transformation improved spectra visual differences, interpretation and models. False negatives and positives prediction error associated with the models developed with raw spectra using the full spectra and spectral data restricted to 1700–1130 cm^-1^ was significantly reduced with Savitzky-Golay first derivative processing.

Overall models developed using raw spectra or spectral data pre-treated with Savitzky-Golay first derivative method with the restricted IR region of 1800–650 cm^-1^ were the best model for discriminating between vaginal swabs containing semen from vaginal swabs with no semen and rayon swabs containing semen only. The models were 100% accurate in assigning the validation samples to their respective groups with zero false negatives/false positives predictions (Table 2).

### Detection of HEC placebo gel

One inherent constraint of using chromatography (LC-MS/MS) for measure of adherence is the limitation that they may only be applied to the active drug arm of a clinical study. Therefore, we evaluated the feasibility of developing a discriminatory model to differentiate a vaginally inserted swab (baseline) from vaginal swabs containing HEC placebo gel.

Two distinct peaks centered at 1540 and 1640 cm^-1^ appear to differentiate baseline vaginal swabs from vaginal swabs containing HEC placebo gel, as shown in S2 Fig (panels a-b). The strongest absorption intensities around peak center 1540 cm^-1^ appear to be the IR peak of interest in discriminating between the spectra of vaginal swabs containing HEC placebo gel from the spectra of baseline swabs, as the latter revealed comparatively higher intensity at this peak. Vibrations resulting to absorption in the IR region of 1640–1540 cm^-1^ are caused by the N-H bend which are associated with amine and amide functional groups. Peaks centered around 1650–1540 cm^-1^ are known to be associated with proteins from biological products present in the vagina [25, 38]. Spectrum of HEC placebo gel is dominated by a strong signal at IR region 1730–1530 cm^-1^ as shown in S2 Fig (Panel b). Presence of placebo gel in the vaginal swabs tended to reduce absorption at this region, which may be attributed to presence of water (about 96% of the gel’s composition). Water is known to be IR active at 1590 cm^-1^ and 1600 cm^-1^ as a result of H-O-H bending mode vibrations.

Although models could be developed using the full IR spectra, models developed using the restricted IR region of 1700–1480 cm^-1^ (S3 Fig) performed better as shown in Table 3. Spectral data pre-treated with Savitzky-Golay first derivative transformation did not appear to have improved model performance. Overall, models developed using the 1700–1480 cm-^1^ IR spectra region performed best in discriminating between baseline swabs and vaginal swabs containing HEC placebo gel with low error. Per Table 3, this model was 97% accurate in identifying new vaginal swabs with HEC gel present with Types I error of 3% and Type II error of 0%.

### Detection of placebo inserts

Five distinct peaks centered at 880, 1000, 1540, 1650 and 2900 cm^-1^ clearly differentiated vaginal swabs from vaginal swabs containing placebo insert, as shown in Fig 4. The spectra in the fingerprint region, from 900 cm^-1^ to 1800 cm^-1^, primarily represent proteins, although the band structure is modified by the presence of nucleic acids and other components.

Absorption intensity around peaks centered at 1650 and1540 cm^-1^ decreased with increasing concentrations of placebo insert on vaginal swabs. Peaks centered around 880, 1000 and 2900 cm^-1^ also showed some visual differences with absorption increasing with increasing insert concentration. However, peaks around 2950–2850 cm^-1^ are usually less useful in structural determination because of the ubiquitous nature of the alkane C-H bonds. Moreover, the absorption could be from background from the rayon swab itself or from the sugar alcohol excipients which make up over 90% w/w composition of the placebo insert. Peaks centered at 880, 1000, 1540 and 1650 cm^-1^ all appear to be the IR peaks of interest in separation of vaginal swabs containing placebo insert from the vaginal swabs with no placebo insert.

All models developed using the full IR spectra, IR region of 1700–780 cm^-1^ and 1200–780 cm^-1^ (S4 Fig) performed accurately in discriminating between baseline vaginal swabs and vaginal swabs containing placebo insert. S2 Table shows the results of validation samples tested against this discriminant model (developed to distinguish between vaginal swabs containing varying concentration of the placebo inserts and baselines and set up to give YES/NO predictions). As shown in the table, the model was accurate in classifying the validation specimens to their respective group. A sample with Mahalanobis distance (M.D.) greater than three is classified as nonvaginal specimen while a specimen with M.D. of less than three is classified as a true member.

To assess feasibility of implementing this discriminatory FTIR model for identification of vaginal swabs containing placebo product in a clinical study setting, the model was further validated using vaginal swabs collected in a clinical study (CONRAD D15-134), in which study participants used a placebo insert vaginally, and vaginal swabs were collected before and after insert use. Table 4 summarizes the FTIR model prediction results from these clinically derived samples, demonstrating that the discriminant model was 100% accurate in identifying baseline and vaginal swabs containing placebo insert collected from participants 15–60 minutes after placebo insert administration. The accuracy of identifying vaginal swabs containing placebo insert reduced to 80% for vaginal swabs collected 24–72 hours after insert use. Together, these results further support the feasibility of using these FTIR based methods for measuring adherence with a vaginal swab in a clinical study using a placebo product.

### Detection of TFV in vaginal swabs

Although the spectrum of the TFV API showed several IR spectroscopic bands as revealed in Fig 5 (panel A), the most important peaks for separation of vaginal swabs containing TFV gel or insert from placebos were found in the IR fingerprint region centered at 830 and 932 cm^-1^(S5 Fig). The IR absorption at the region 930–830 cm^-1^ could be attributed to the N-H wagging, and C-H out-of-plane deformation bands occuring at IR region of 900–600 cm^-1^. These findings are also similar to reported findings of Zidan et al., in which raw spectra of TFV was found to produce FTIR spectroscopic bands corresponding to several of its functional groups [41].

PCA models developed using the IR fingerprint region of 930–830 cm^−1^ revealed that the best model for clear and unambigious separation of vaginal swabs containing active gel from placebo or baseline could be achieved using the IR region of 930–830 cm^-1^ (S6 Fig, Panel C). We observed that the first 2 pc’s explained about 99% of spectra variation. A similar result was observed for models designed for separation of vaginal swabs containing active inserts and placebo inserts (data not shown).

Table 5 shows the results of validation samples tested to assess the feasibility of using FTIR coupled with the discriminant model to identify and discriminate between samples of vaginal swabs (baseline), vaginal swabs containing placebo inserts, and vaginal swabs containing TFV. As shown in the table, the model was accurate in classifying the validation specimens to their respective group.

### Comparison of FTIR to other methods used to measure adherence of microbicide products

Because of its sensitivity and repeatability, quantitating systemic or tissue anti-retroviral (ARV) levels through LC-MS/MS is currently viewed as the gold standard and most reliable measure of adherence in microbicide field. However, its usage is inherently constrained by the limitations of cost, time-intensive sample preparations, high technical skill requirement, and its applicability to only the active treatment group in clinical trials. Although the sensitivity of an IR spectroscopic technique may be lower than LC-MS/MS, IR techniques present attractive features such as being fast, solvent- and sample preparation-free, cost-effective, easy-to-use and even portable for potential use in-field or at a clinical site. As demonstrated in this report, it also may be applicable as a measure of adherence for both active and placebo groups. If properly set up, FTIR analysis will take less time to provide a simple clinical laboratory test that could be performed in the field. This could enable real-time interventions for supporting and improving participant adherence in clinical trials.

Like LC-MS/MS, the FTIR analysis methods presented here are designed to detect biomarkers of protocol compliance, i.e., vaginally inserting a swab and semen exposure, and also to detect product use, i.e., topical, vaginal application of TFV and placebo. In a previous CONRAD study investigating adherence biomarkers from vaginally used TFV 1% gel applicators obtained from a large HIV prevention trial in Africa, we determined that residual TFV gel on vaginally used, gel expelled applicators can be swabbed and measured directly via FTIR and also found out that FTIR predictions was close to 90% in agreement with LC-MS/MS reference values (manuscript in preparation). In another study, CONRAD D15-135 focused on clinical assessment of the FTIR methods and other novel, excipient-based objective adherence measures for four placebo vaginal dosage platforms, FTIR results were similar and consistent to the excipient-based assays developed and the study provided feasibility data that FTIR could be used to determine vaginal placebo product use (manuscript under review).

## Conclusion

We have demonstrated the proof of concept that IR spectroscopy coupled with MVDA modeling can detect and discriminate between placebo and active vaginal microbicide products and identify semen exposure and vaginal use using a self-collected vaginal swab. To the best of our knowledge, this is the first attempt to use a FTIR and chemometric approach to detect microbicide products and other biological fluids in vaginal swabs as a measure of adherence in clinical settings. Given the portability of the FTIR equipment used, the ability to program the equipment to provide simple “yes” or “no” prediction readouts after direct and non-destructive analysis of a vaginal swab, we believe the FTIR technology has great potential to monitor adherence in clinical or field settings. With proper calibration models and control of other factors (such as variations in instruments, environment, and material handling), it may be applied as a quick screening method for real-time on-site adherence monitoring to many drug products and biological fluids (S7 Fig).

These methods have since been validated in a clinical study of placebo vaginal products (gel, film, insert) used alone or prior to unprotected sex (CONRAD 135, manuscript in preparation), and are being implemented as part of a composite adherence measure in a placebo product acceptability trial in South Africa and Zimbabwe (The Quatro Study, #NCT02602366).

Although initially developed to identify semen exposure and concurrent presence of TFV in the vagina, the method can be further refined to detect other antiviral compounds in multiple compartments.

## Supporting information

S1 Table Validation of discriminant model for identification of vaginally inserted swabs from non-vaginal swabs. (DOCX)

S2 Table Validation of discriminant model for identification of vaginal swabs containing placebo inserts. (DOCX)

S1 Fig ATR-FTIR spectra comparison of semen and vaginal fluid (panel A) and score plot showing separation of spectra of vaginal secretion swabs (no semen) from vaginal swabs containing semen and rayon swab containing semen only (panel B). (TIF)

S2 Fig Representative IR (1700–1480 cm^-1^) raw spectra (panel A) and Savitzky-Golay first derivative transformed spectra (panel B) of vaginal swabs and vaginal swabs containing HEC placebo gel. (TIF)

S3 Fig Score plots showing separation of spectra of vaginal swabs (no HEC gel) from vaginal swabs containing HEC gel. (TIF)

S4 Fig Score plots showing separation of spectra of vaginal swabs (baseline) from vaginal swabs containing placebo inserts prepared at varying concentrations. (TIF)

S5 Fig Spectra showing vaginal swabs containing TFV gel of varying concentration (panel A) and separation from vaginal swabs containing placebo gel or no product (panel B). (TIF)

S6 Fig PCA score plots showing separation of vaginal swabs containing TFV gel from vaginal swabs containing placebo and no product (Baseline) using IR spectra region 4000–650 cm^-1^ (panel A), 1800–650 cm^-1^(panel B) and 930–830 cm^-1^ (panel C). (TIF)

S7 Fig Schematic of expected workflow for an FTIR quick screening method for real-time on-site adherence monitoring. (TIF)
