# Supplementary material for: Rapid measures of user’s adherence to vaginal drug products using attenuated total reflectance Fourier transform infrared spectroscopy (ATR-FTIR) and multivariate discriminant techniques
Source: PLoS One. 2018 May 25;13(5):e0197906. doi: 10.1371/journal.pone.0197906 (PMC5969765; doi:10.1371/journal.pone.0197906)
Supplement: S1 Table — (DOCX) [file pone.0197906.s001.docx]

S1 Table. Validation of discriminant model for identification of vaginally inserted swabs from non-vaginal swabs

| **Sample** | **Prediction^a^** | **Mahalanobis Distance^b^** |
| --- | --- | --- |
| Dry Rayon 1 | No | 676.38 |
| Dry Rayon 2 | No | 538.80 |
| Dry Rayon 3 | No | 344.37 |
| Dry Rayon 4 | No | 431.88 |
| VAGINAL SIMULANT FLUID 1 | No | 16.52 |
| VAGINAL SIMULANT FLUID 2 | No | 18.25 |
| VAGINAL SIMULANT FLUID 3 | No | 11.08 |
| VAGINAL SIMULANT FLUID 4 | No | 15.70 |
| Water 1 | No | 15.75 |
| Water 2 | No | 17.13 |
| Water 3 | No | 26.51 |
| Water 4 | No | 36.71 |
| VAGINAL SWAB 1 | Yes | 0.72 |
| VAGINAL SWAB 2 | Yes | 0.59 |
| VAGINAL SWAB 3 | Yes | 0.83 |
| VAGINAL SWAB 4 | Yes | 1.01 |
| VAGINAL SWAB 5 | Yes | 1.04 |
| VAGINAL SWAB 6 | Yes | 1.09 |
| VAGINAL SWAB 7 | Yes | 0.84 |
| VAGINAL SWAB 8 | Yes | 0.67 |

^a^ Yes/no prediction based on model asking the question: Is this a vaginally-inserted swab?

^b^ A specimen with Mahalanobis distance (M.D.) greater than 3 is classified as a nonvaginal specimen while a specimen with M.D of less than 3 is classified as a true member
