# Supplementary material for: Rapid measures of user’s adherence to vaginal drug products using attenuated total reflectance Fourier transform infrared spectroscopy (ATR-FTIR) and multivariate discriminant techniques
Source: PLoS One. 2018 May 25;13(5):e0197906. doi: 10.1371/journal.pone.0197906 (PMC5969765; doi:10.1371/journal.pone.0197906)
Supplement: S2 Table — (DOCX) [file pone.0197906.s002.docx]

S2 Table. Validation of discriminant model for identification of vaginal swabs containing placebo inserts

| **Sample** | **Prediction^a^** | **Mahalanobis Distance^b^** |
| --- | --- | --- |
| Placebo insert -0.1 g/mL | Yes | 1.279501137 |
| Placebo insert -0.1 g/mL | Yes | 1.0234527 |
| Placebo insert -0.1 g/mL | Yes | 0.935071759 |
| Placebo insert -0.1 g/mL | Yes | 1.743954884 |
| Placebo insert -0.25 g/mL | Yes | 1.230846227 |
| Placebo insert -0.25 g/mL | Yes | 0.681501961 |
| Placebo insert -0.25 g/mL | Yes | 1.075158768 |
| Placebo insert -0.25 g/mL | Yes | 1.049142912 |
| Placebo insert -0.4 g/mL | Yes | 0.732392727 |
| Placebo insert -0.4 g/mL | Yes | 0.820367802 |
| Placebo insert -0.4 g/mL | Yes | 1.005141013 |
| Placebo insert -0.4 g/mL | Yes | 0.957735934 |
| Vaginal swab (no placebo insert) 1 | No | 8.596828768 |
| Vaginal swab (no placebo insert) 2 | No | 24.42152554 |
| Vaginal swab (no placebo insert) 3 | No | 6.509212919 |
| Vaginal swab (no placebo insert) 4 | No | 6.417141335 |
| Vaginal swab (no placebo insert) 5 | No | 9.996869784 |
| Vaginal swab (no placebo insert) 6 | No | 7.746259412 |
| Vaginal swab (no placebo insert) 7 | No | 8.650532111 |
| Vaginal swab (no placebo insert) 8 | No | 6.773756989 |
| Vaginal swab (no placebo insert) 9 | No | 3.894429854 |
| Vaginal swab (no placebo insert) 10 | No | 30.21004728 |
| Vaginal swab (no placebo insert) 11 | No | 4.248384362 |
| Vaginal swab (no placebo insert) 12 | No | 33.24067951 |

^a^ Yes/no prediction based on model asking the question: Is placebo insert present?

^b^ A specimen with Mahalanobis distance (M.D.) greater than 3 is classified as NO for presence of placebo insert, while a specimen with M.D of less than 3 is classified as a Yes member
