# Supplementary figures and images for: Rapid measures of user’s adherence to vaginal drug products using attenuated total reflectance Fourier transform infrared spectroscopy (ATR-FTIR) and multivariate discriminant techniques
Source: PLoS One. 2018 May 25;13(5):e0197906. doi: 10.1371/journal.pone.0197906 (PMC5969765; doi:10.1371/journal.pone.0197906)

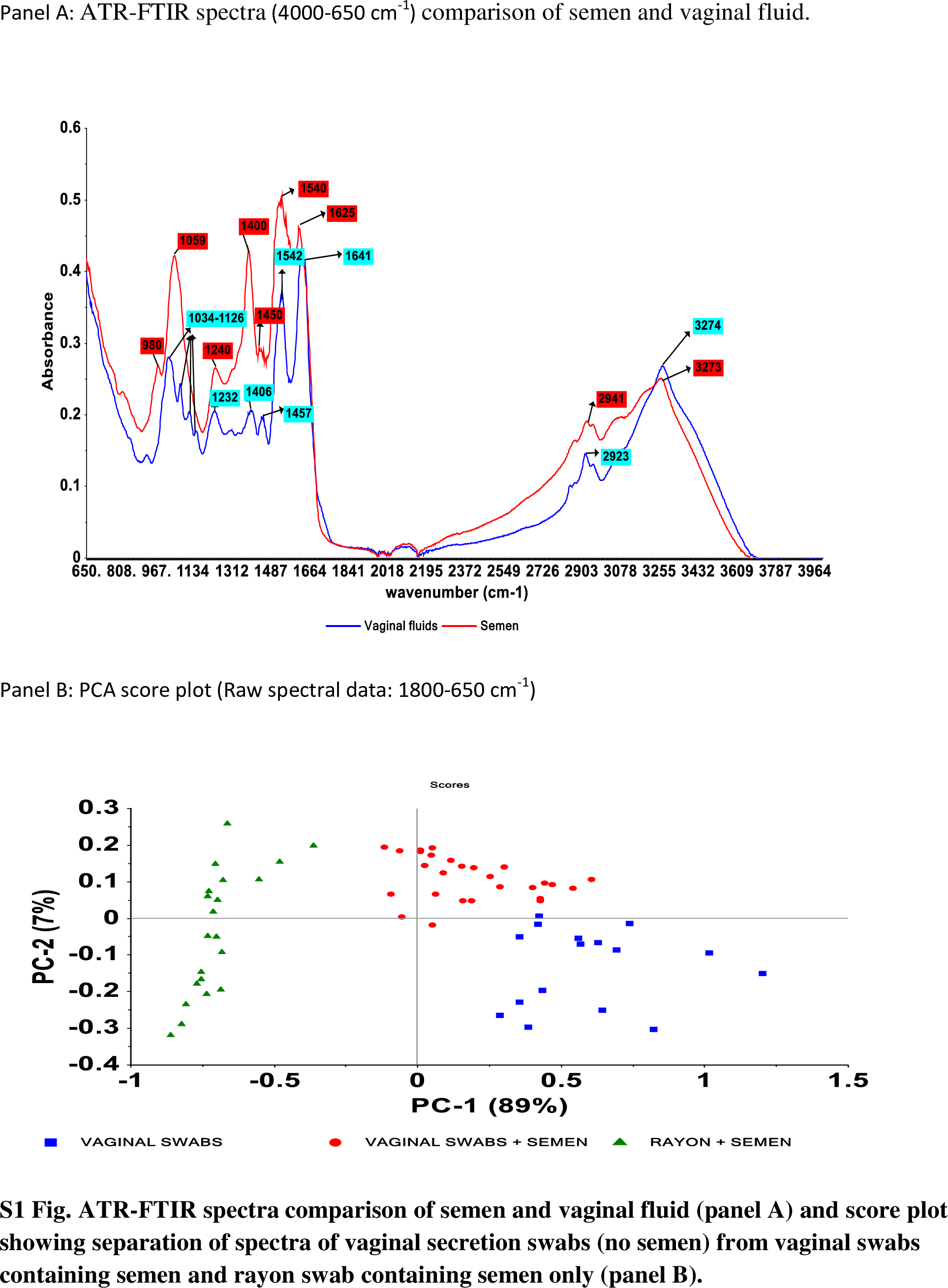

Supplement: S1 Fig — (TIF) [file pone.0197906.s003.tif]

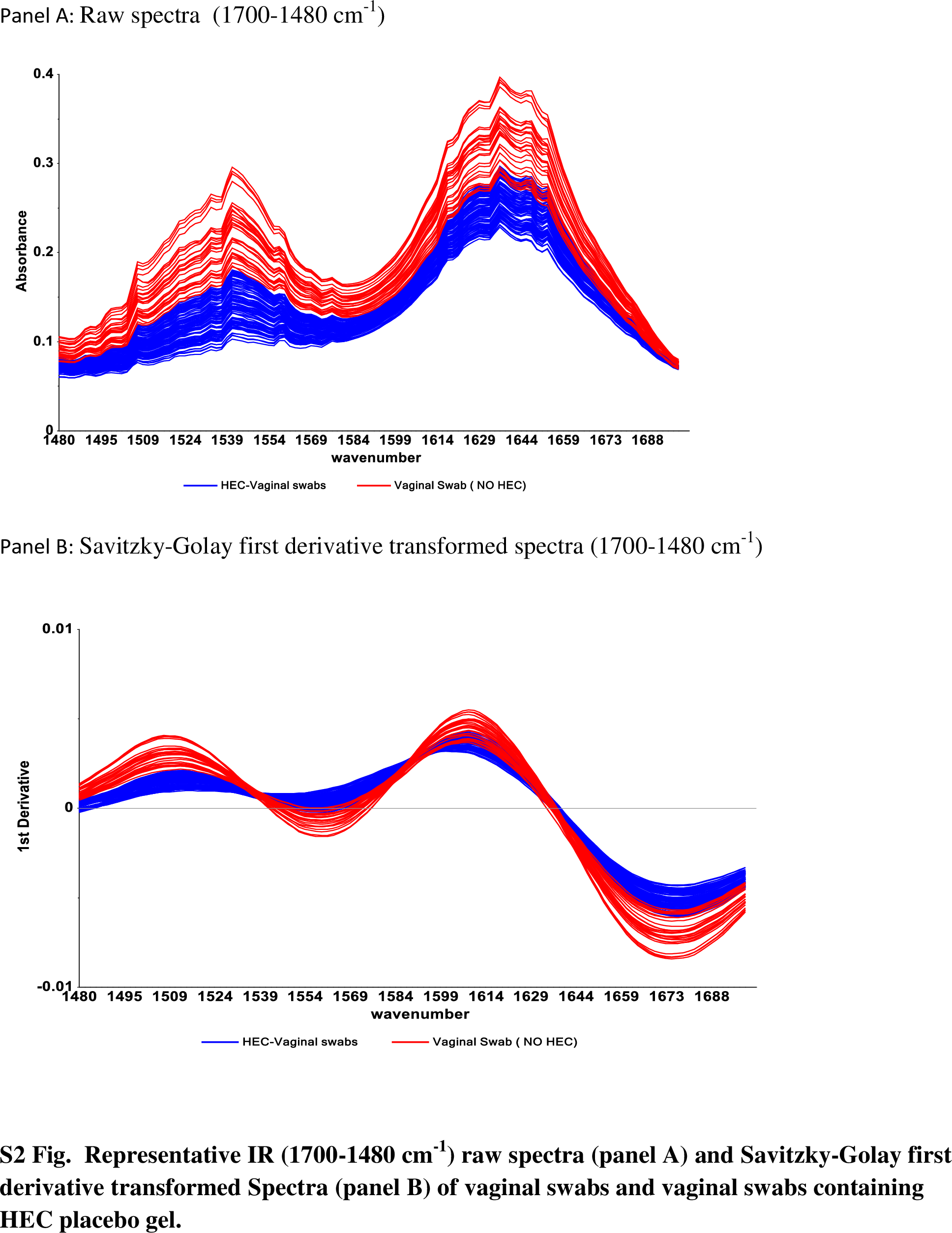

Supplement: S2 Fig — (TIF) [file pone.0197906.s004.tif]

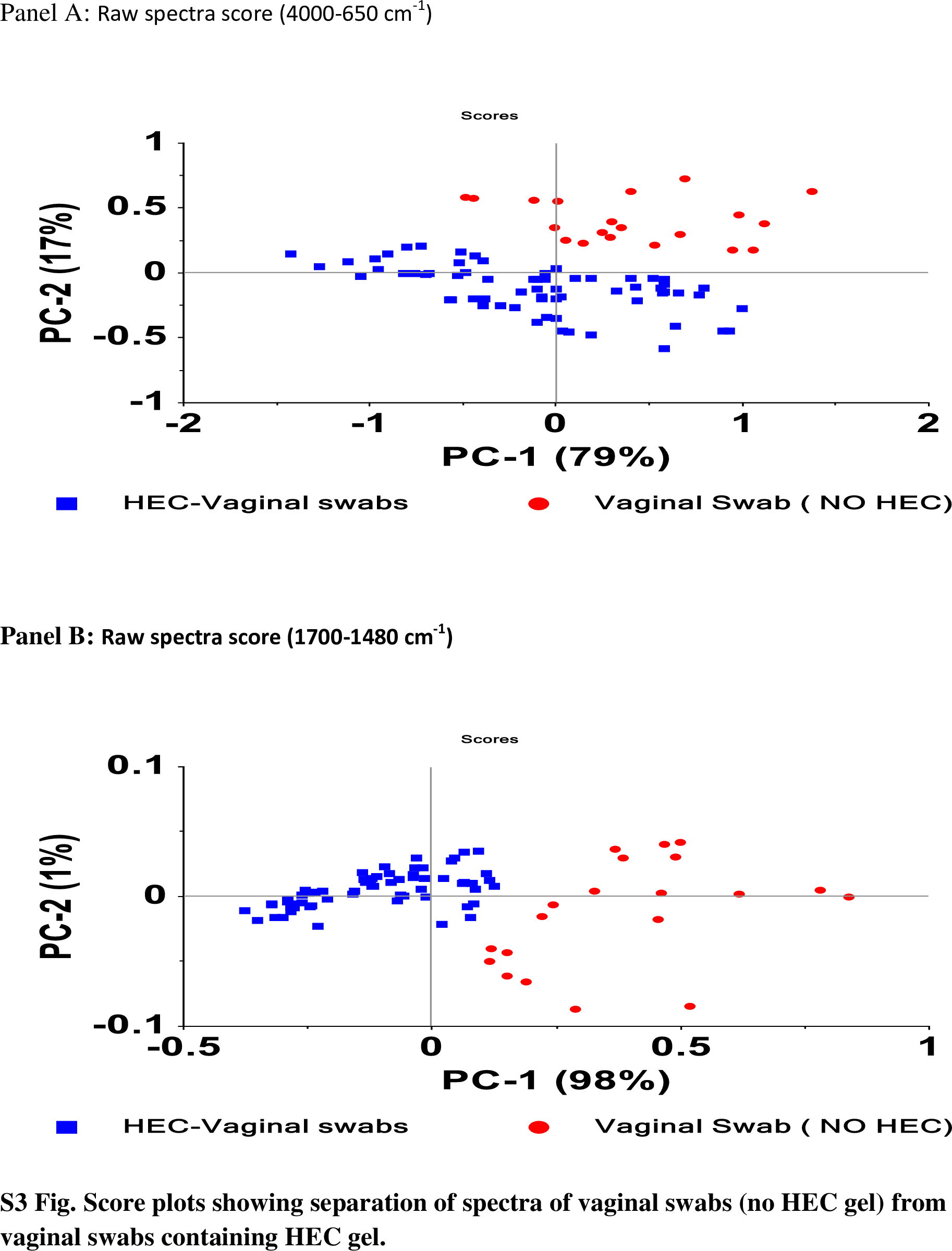

Supplement: S3 Fig — (TIF) [file pone.0197906.s005.tif]

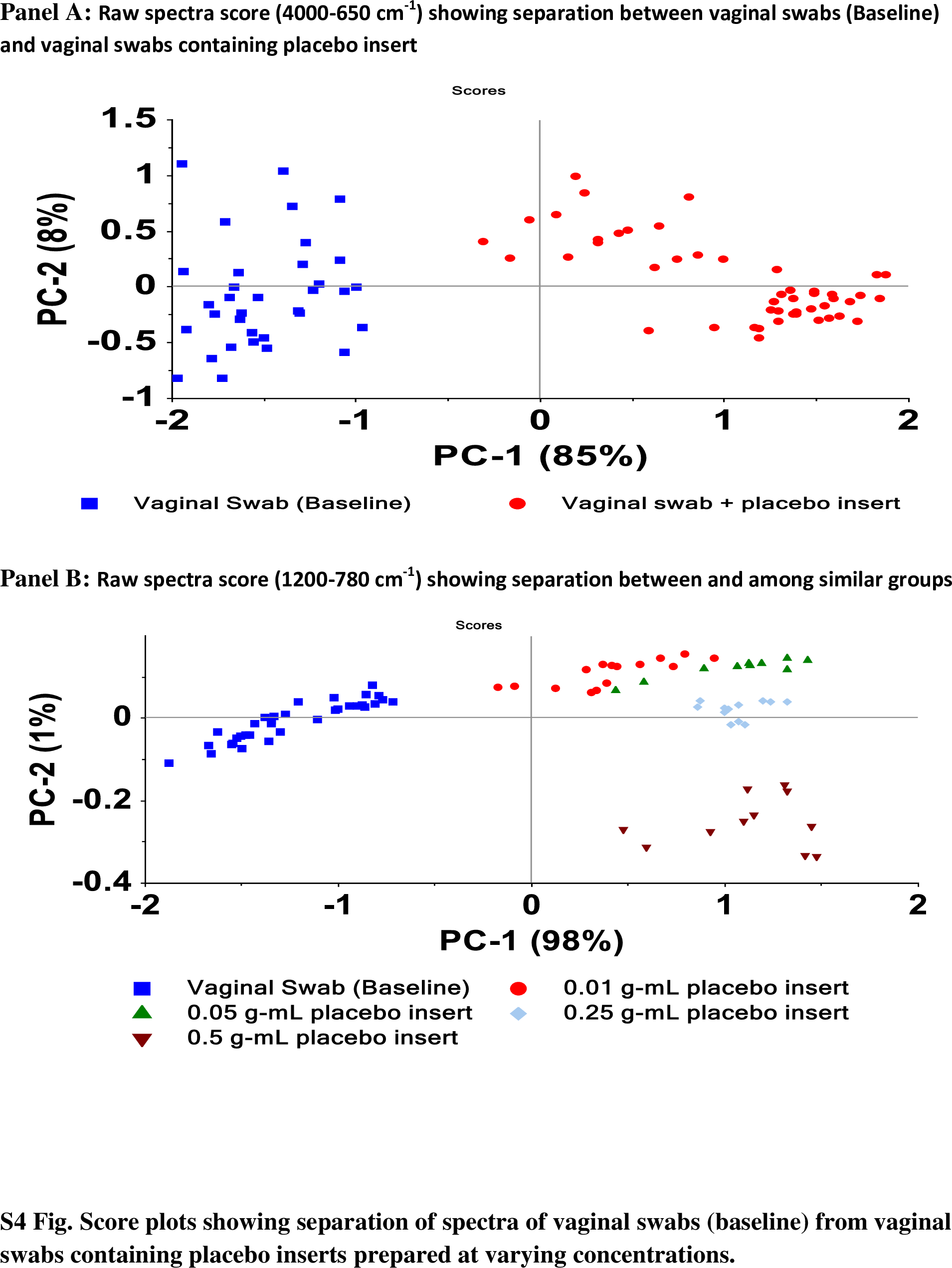

Supplement: S4 Fig — (TIF) [file pone.0197906.s006.tif]

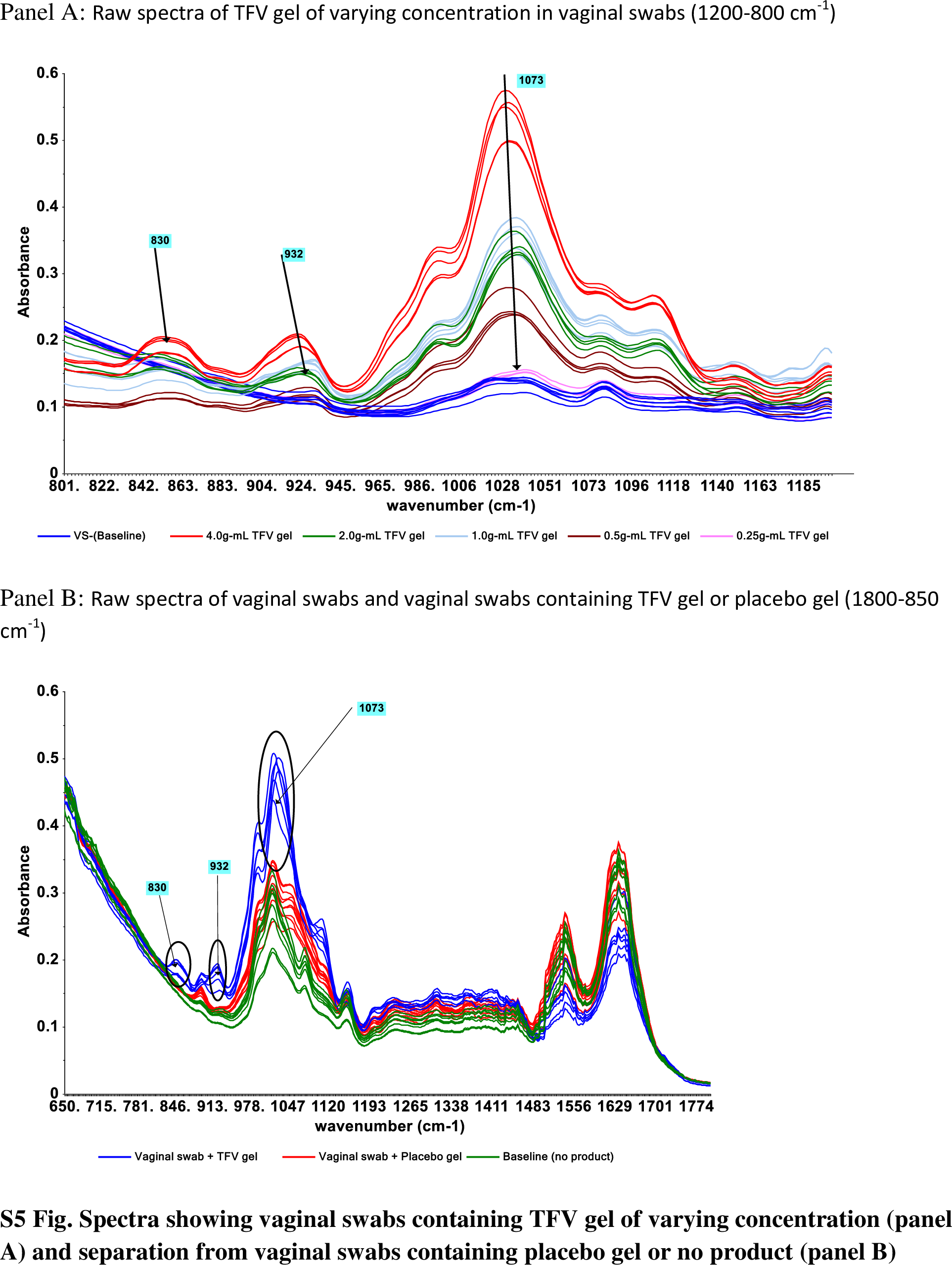

Supplement: S5 Fig — (TIF) [file pone.0197906.s007.tif]

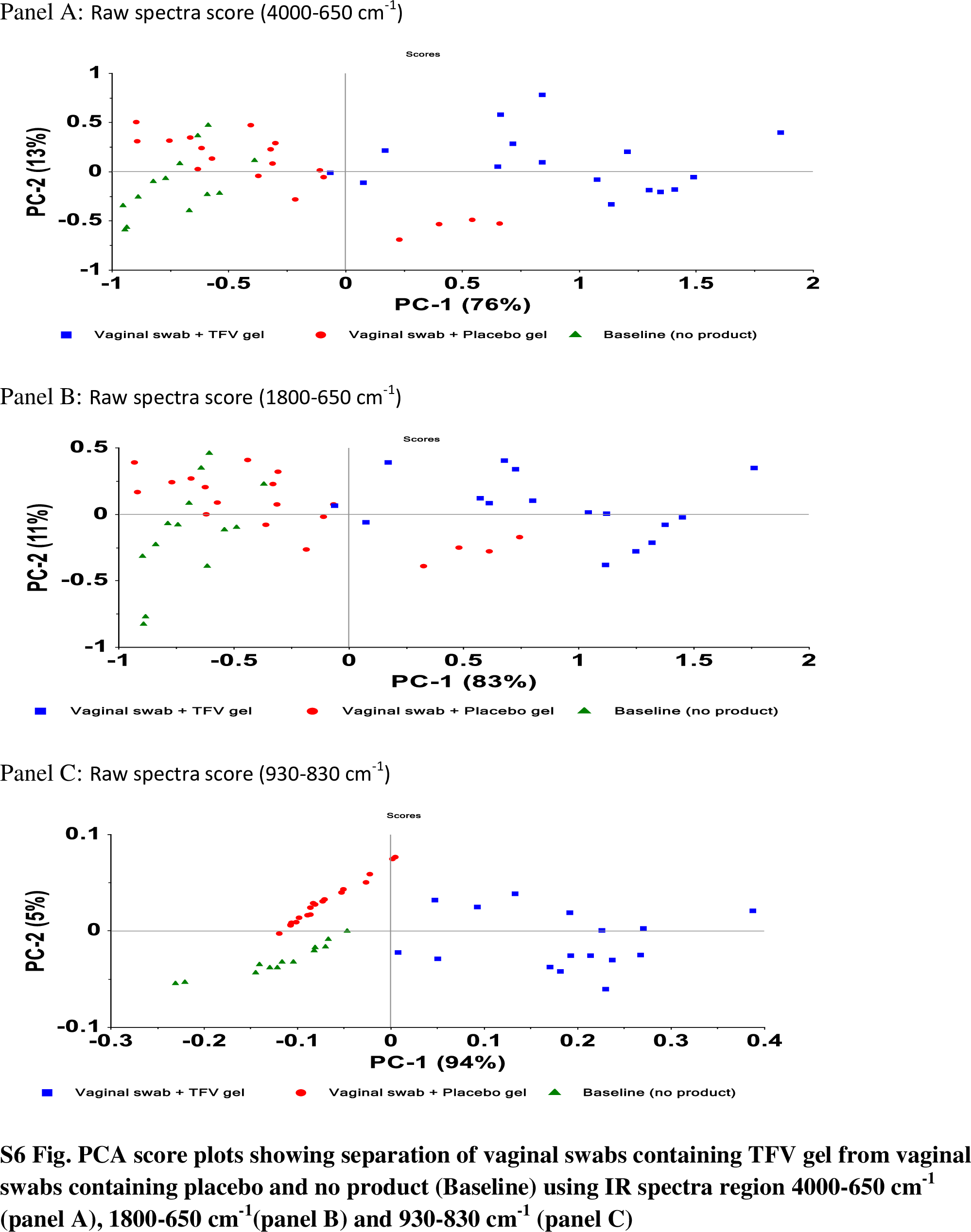

Supplement: S6 Fig — (TIF) [file pone.0197906.s008.tif]

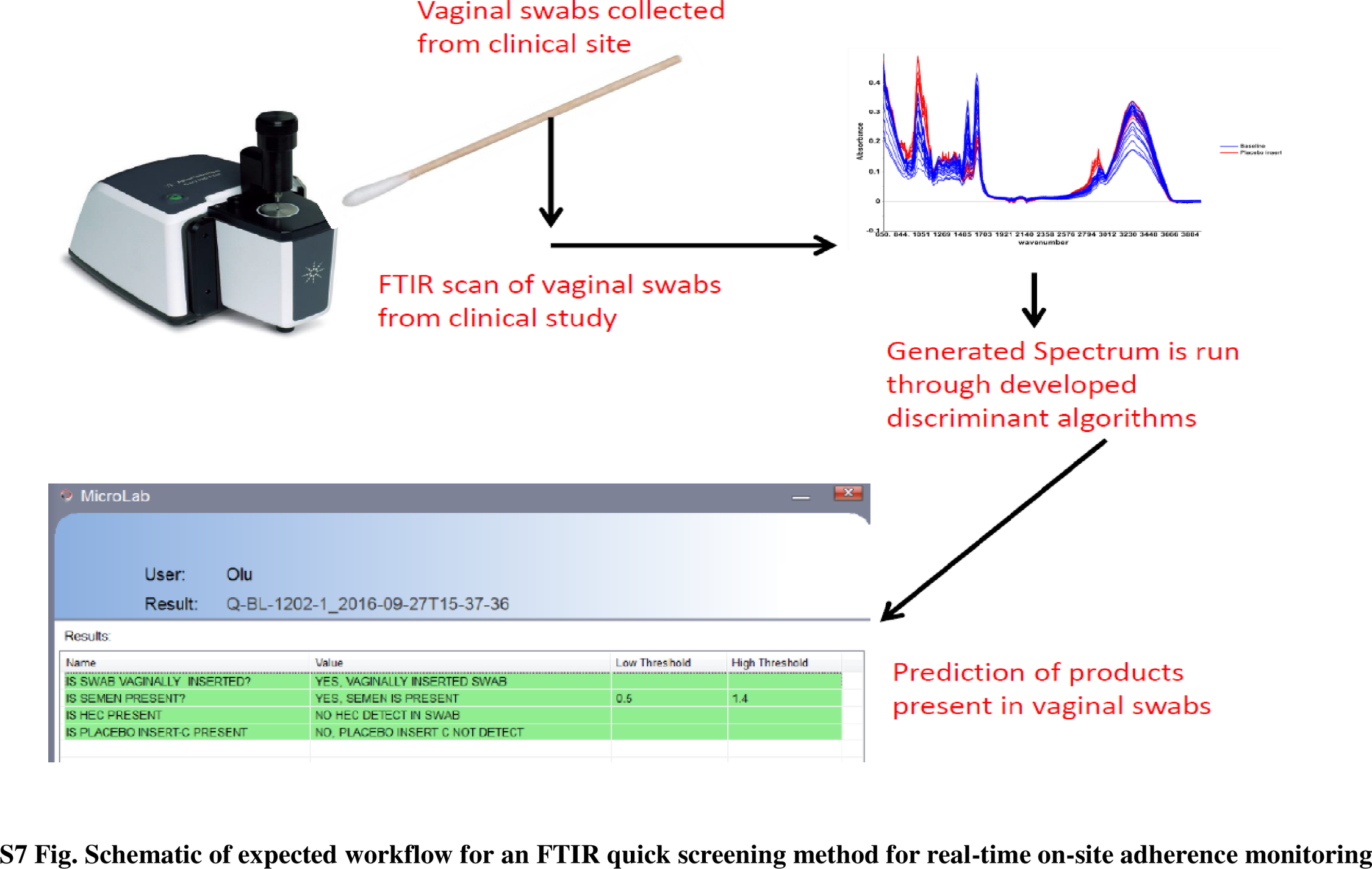

Supplement: S7 Fig — (TIF) [file pone.0197906.s009.tif]
